# Editorial: The Androgen Receptor in Breast Cancer

**DOI:** 10.3389/fendo.2020.636480

**Published:** 2021-01-15

**Authors:** Anna Tesei, Gabriella Castoria

**Affiliations:** ^1^Biosciences Laboratory, Istituto Scientifico Romagnolo per lo Studio e la Cura dei Tumori (IRST), Istituto di Ricovero e Cura a Carattere Scientifico (IRCCS), Meldola, Italy; ^2^Dipartimento di Medicina di Precisione, Università della Campania “L. Vanvitelli”, Naples, Italy

**Keywords:** androgen receptor, breast cancer, triple-negative breast cancers, anti-androgen therapy, non-genomic effects of the androgen receptor

Androgen receptor (AR) is often overexpressed in breast cancer (BC) (1–3) and is the prevalent sex steroid receptor in “*in situ*,” invasive and metastatic BC (4–8). Moreover, up to 30% of triple negative breast cancers (TNBCs), the most aggressive forms of BC, express AR, which is also the only sex steroid receptor detectable in BC metastases and in metastatic lesions resected at autopsy (9–11). Although several findings support a role for androgen/AR axis in BC, its involvement in the pathogenesis and progression of this cancer remains debated, and the predictive and prognostic role of AR in TNBCs is even less clear.

By gene expression analysis of several BC data sets, Lehmann and colleagues identified six different TNBC subtypes (12). The luminal AR (LAR) subtype differs from the others in gene expression profile and ontology. Analysis of gene expression in the LAR subtype was consistent with a subset of estrogen receptor (ER)^−^/AR^+^ BCs (also known as molecular apocrine tumors), which express genes commonly detected in luminal tumors, such as *XPB-1*, *SCUBE2*, *SPDEF*, and *FOXA1* (13, 14). These findings, together with the observed sensitivity of LAR-derived cells to the anti-androgen bicalutamide, strengthened the concept that AR is a key component of the LAR-TNBC subtype and that AR-targeted therapies may be effective against BCs expressing this hormone receptor (12). The benefits of anti-androgen treatment for AR-expressing BCs have since become the subject of an ongoing discussion among oncologists.

The historical success of AR targeting in prostate cancer (PC) has provided a proof-of-principle for its application in BC therapy. Specifically, the development of new, more effective, and safer AR-targeted agents has once again opened up the debate on whether or not to use these compounds in BCs and especially in TNBCs, for which no specific treatment guidelines are available and systemic chemotherapy remains almost the only option in both early and advanced stages of the disease. Anti-androgen therapies showed promising results in preclinical models of BCs (15–18) and several clinical trials have evaluated the efficacy of this approach in patients with AR^+^ TNBC; very encouraging data from three trials investigating the use of bicalutamide, abiraterone acetate, and enzalutamide report clinical benefit in about one in five patients (19–21). Of note, as in PC, the onset of a hormone-refractory state following prolonged use of selective androgen receptor modifiers is expected, and the findings from clinical trials make a case for identifying predictive response factors and therapeutic combinations in order to translate advancements in the field of BC into clinical practice.

The complexity of AR action is further underscored by studies on non-genomic actions mediated by the hormone receptor in BCs. The ligand-bound receptor interacts with various signaling effectors in the extranuclear compartment of target cells. Depending on cell type and hormone concentration, androgen-triggered association of AR with the tyrosine kinase Src activates Src-dependent signaling pathway, leading to cell proliferation, while hormone-stimulated interaction between AR and filamin A controls machinery driving cell motility and invasiveness (22). Of note, the role of Src and PI3K-dependent signaling pathways in promoting invasiveness triggered by androgen/AR axis has been recently dissected in various TNBC cell subtypes (23). These and other similar findings might pave the way toward the identification of biomarkers predictive of TNBC malignancy and the exploration of novel compounds for clinical application.

In this Research Topic, we have invited leading groups to contribute review and original research articles on the role of AR in BC. Three reviews within this Research Topic address some of the questions still pending and identify challenges in the field, with the aim of re-examining the intricate molecular landscape of AR and offering new clues as to its prognostic and therapeutic value in BCs. Bleach and Mcllroy discuss the complexity of AR-driven mechanisms within the context of our current knowledge of steroid intracrinology in BC. The paper describes the action of key mediators of transcriptional or non-transcriptional AR-induced events in BC, with particular emphasis on the role of intracrine ligands and their fluctuations. Interestingly, the review outlines the role of AR/ERα crosstalk occurring at transcriptional level in BCs, together with factors that modulate their interaction. The simultaneous targeting of AR and ERα is an intriguing proposition and might provide a valid approach to overcome therapeutic resistance in BC.

Over the last decade, conflicting evidence has been reported on the role of AR in BCs. Giovannelli et al. review major findings on the biology of AR in BC, with a focus on its prognostic and predictive role in TNBCs. The authors discuss the development of future therapeutic strategies in this BC subtype in light of the group’s most recent discoveries obtained using new site-specific compounds that specifically perturb the non-genomic mechanism mediated by AR in preclinical models of TNBCs (23). Inhibition of this functional action of AR may enhance the effect of traditional AR transcriptional inhibitors and warrant further investigation in clinical models of TNBCs.

Lastly, Chen et al. explore the role of AR and AR-related mechanisms in various BC subtypes, with a focus on experimental limitations and issues encountered in translating research from bench to bedside. The review spotlights the need to develop more appropriate AR detection methods and to identify new markers of AR responsiveness for better stratification of patients who might benefit from AR-targeted treatments.

BC therapy has evolved over the years from surgery, chemotherapy, radiation therapy, and hormone therapy to combinatorial treatments (24, 25). However, many patients, particularly TNBC patients, still experience a very high rate of recurrence, regardless of subtype. Because of the paucity of available therapies for as well as the intrinsic insensitivity of TNBCs to radiation therapy (26), the development of new radiosensitizing strategies is needed to improve clinical outcomes. The research article by Michmerhuizen et al. reports promising data on the effects of seviteronel, a non-steroidal selective CYP17-lyase inhibitor that blocks AR activation. Although the compound exhibits a limited effect in monotherapy, it shows great promise in radiosensitizing AR^+^ TNBCs in preclinical models. These findings may help expand the toolkit of radiotherapists and oncologists.

Differences in BC outcome among specific patient groups based on age, ethnicity, disability, and other factors are widely recognized. By comparing the biological characteristics of Tanzanian and Italian BC patients matched for date of and age at diagnosis, the research article by Bravaccini et al. examines the role of AR as a prognostic marker in a Sub-Saharan African setting, where access to healthcare and cancer prevention services is extremely poor. The paper reports that TNBCs analyzed among the Tanzanian population expressed very high AR levels, opening up new therapeutic perspectives in this low-income country.

In recent years, precision medicine strategies have yielded very encouraging results that lay the groundwork for more personalized diagnosis and treatment of AR-expressing BCs. However, much work is still needed to further explore the molecular and clinical aspects of this disease. The efforts of multidisciplinary teams combining molecular endocrinologists, oncologists, clinicians, and radiotherapists are expected to deliver high-quality research and therapeutic advances in the global battle against BC.

## Author Contributions

AT and GC wrote, reviewed, and approved the content of this editorial. All authors contributed to the article and approved the submitted version.

## Conflict of Interest

The authors declare that the research was conducted in the absence of any commercial or financial relationships that could be construed as a potential conflict of interest.

## References

[1] Niemeier LA Dabbs DJ Beriwal S Striebel JM Bhargava R Androgen receptor in breast cancer: Expression in estrogen receptor-positive tumors and in estrogen receptor-negative tumors with apocrine differentiation. Mod Pathol (2010) 23:205–12. 10.1038/modpathol.2009.159 19898421

[2] Hickey TE Robinson JLL Carroll JS Tilley WD Minireview: The androgen receptor in breast tissues: Growth inhibitor, tumor suppressor, oncogene? Mol Endocrinol (2012) 26:1252–67. 10.1210/me.2012-1107 PMC3404296 22745190

[3] Hu R Dawood S Holmes MD Collins LC Schnitt SJ Cole K Androgen receptor expression and breast cancer survival in postmenopausal women. Clin Cancer Res (2011) 17:1867–74. 10.1158/1078-0432.CCR-10-2021 PMC3076683 21325075

[4] Peters AA Buchanan G Ricciardelli C Bianco-Miotto T Centenera MM Harris JM Androgen receptor inhibits estrogen receptor-α activity and is prognostic in breast cancer. Cancer Res (2009) 69:6131–40. 10.1158/0008-5472.CAN-09-0452 19638585

[5] Castellano I Allia E Accortanzo V Vandone AM Chiusa L Arisio R Androgen receptor expression is a significant prognostic factor in estrogen receptor positive breast cancers. Breast Cancer Res Treat (2010) 124:607–17. 10.1007/s10549-010-0761-y 20127405

[6] Buchanan G Birrell SN Peters AA Bianco-Miotto T Ramsay K Cops EJ Decreased androgen receptor levels and receptor function in breast cancer contribute to the failure of response to medroxyprogesterone acetate. Cancer Res (2005) 65:8487–96. 10.1158/0008-5472.CAN-04-3077 16166329

[7] Lønning PE Additive endocrine therapy for advanced breast cancer back to the future. Acta Oncol (Madr) (2009) 48:1092–101. 10.3109/02841860903117816 19863216

[8] Gucalp A Traina TA Triple-negative breast cancer: Role of the androgen receptor. Cancer J (2010) 16:62–5. 10.1097/PPO.0b013e3181ce4ae1 20164692

[9] Lea OA Kvinnsland S Thorsen T Improved Measurement of Androgen Receptors in Human Breast Cancer. Cancer Res (1989) 49:7162–7. 2582456

[10] Park S Koo J Park HS Kim JH Choi SY Lee JH Expression of androgen receptors in primary breast cancer. Ann Oncol (2009) 21:488–92. 10.1093/annonc/mdp510 19887463

[11] Cimino-Mathews A Hicks JL Illei PB Halushka MK Fetting JH De Marzo AM Androgen receptor expression is usually maintained in initial surgically resected breast cancer metastases but is often lost in end-stage metastases found at autopsy. Hum Pathol (2012) 43:1003–11. 10.1016/j.humpath.2011.08.007 PMC3328602 22154362

[12] Lehmann BD Bauer JA Chen X Sanders ME Chakravarthy AB Shyr Y Identification of human triple-negative breast cancer subtypes and preclinical models for selection of targeted therapies. J Clin Invest (2011) 121:2750–67. 10.1172/JCI45014 PMC3127435 21633166

[13] Farmer P Bonnefoi H Becette V Tubiana-Hulin M Fumoleau P Larsimont D Identification of molecular apocrine breast tumours by microarray analysis. Oncogene (2005) 24:4660–71. 10.1038/sj.onc.1208561 15897907

[14] Doane AS Danso M Lal P Donaton M Zhang L Hudis C An estrogen receptor-negative breast cancer subset characterized by a hormonally regulated transcriptional program and response to androgen. Oncogene (2006) 25:3994–4008. 10.1038/sj.onc.1209415 16491124

[15] Naderi A Chia KM Liu J Synergy between inhibitors of androgen receptor and MEK has therapeutic implications in estrogen receptor-negative breast cancer. Breast Cancer Res (2011) 13:R36. 10.1186/bcr2858 21457548 PMC3219199

[16] Nahleh Z Androgen receptor as a target for the treatment of hormone receptor-negative breast cancer: an unchartered territory. Futur Oncol (2008) 4:15–21. 10.2217/14796694.4.1.15 18240997

[17] Ni M Chen Y Lim E Wimberly H Bailey ST Imai Y Targeting Androgen Receptor in Estrogen Receptor-Negative Breast Cancer. Cancer Cell (2011) 20:119–31. 10.1016/j.ccr.2011.05.026 PMC3180861 21741601

[18] Jing Y Cui D Guo W Jiang J Jiang B Lu Y Activated androgen receptor promotes bladder cancer metastasis *via* Slug mediated epithelial-mesenchymal transition. Cancer Lett (2014) 348:135–45. 10.1016/j.canlet.2014.03.018 24662746

[19] Gucalp A Tolaney S Isakoff SJ Ingle JN Liu MC Carey LA Phase II trial of bicalutamide in patients with androgen receptor-positive, estrogen receptor-negative metastatic breast cancer. Clin Cancer Res (2013) 19:5505–12. 10.1158/1078-0432.CCR-12-3327 PMC4086643 23965901

[20] Bonnefoi H Grellety T Tredan O Saghatchian M Dalenc F Mailliez A A phase II trial of abiraterone acetate plus prednisone in patients with triple-negative androgen receptor positive locally advanced or metastatic breast cancer (UCBG 12-1). Ann Oncol (2016) 27:812–8. 10.1093/annonc/mdw067 27052658

[21] Traina TA Miller K Yardley DA Eakle J Schwartzberg LS O’Shaughnessy J Enzalutamide for the treatment of androgen receptor-expressing triple-negative breast cancer. J Clin Oncol (2018) 36:884–90. 10.1200/JCO.2016.71.3495 PMC5858523 29373071

[22] Castoria G Auricchio F Migliaccio A Extranuclear partners of androgen receptor: at the crossroads of proliferation, migration, and neuritogenesis. FASEB J (2017) 31:1289–300. 10.1096/fj.201601047R 28031322

[23] Giovannelli P Di Donato M Auricchio F Castoria G Migliaccio A Androgens Induce Invasiveness of Triple Negative Breast Cancer Cells Through AR/Src/PI3-K Complex Assembly. Sci Rep (2019) 9(1):4490. 10.1038/s41598-019-41016-4 30872694 PMC6418124

[24] Abe O Abe R Enomoto K Kikuchi K Koyama H Masuda H Effects of radiotherapy and of differences in the extent of surgery for early breast cancer on local recurrence and 15-year survival: An overview of the randomised trials. Lancet (2005) 366:2087–106. 10.1016/S0140-6736(05)67887-7 16360786

[25] Clarke M Meta-analyses of adjuvant therapies for women with early breast cancer: The Early Breast Cancer Trialists’ Collaborative Group overview. Ann Oncol (2006) 17(Supplement 10):x59–62. 10.1093/annonc/mdl238 17018753

[26] Kyndi M Sørensen FB Knudsen H Overgaard M Nielsen HM Overgaard J Estrogen receptor, progesterone receptor, HER-2, and response to postmastectomy radiotherapy in high-risk breast cancer: the danish breast cancer cooperative group. JCO (2008) 26:1419–26. 10.1200/JCO.2007.14.5565 18285604

